# FAM20B-Catalyzed Glycosylation Regulates the Chondrogenic and Osteogenic Differentiation of the Embryonic Condyle by Controlling IHH Diffusion and Release

**DOI:** 10.3390/ijms26094033

**Published:** 2025-04-24

**Authors:** Xiaoyan Chen, Han Liu, Yuhong Huang, Leilei Li, Xuxi Jiang, Bo Liu, Nan Li, Lei Zhu, Chao Liu, Jing Xiao

**Affiliations:** 1 Department of Oral Pathology, School of Stomatology, Dalian Medical University, Dalian 116044, China; dlcxy5285@163.com (X.C.); dlliuhan@163.com (H.L.);; 2 Academician Laboratory of Immunology and Oral Development & Regeneration, Dalian Medical University, Dalian 116044, China; 3 Institute for Genome Engineered Animal Models of Human Diseases, Dalian Medical University, Dalian 116044, China

**Keywords:** FAM20B, proteoglycan, glycosaminoglycan chain, temporomandibular joint, endochondral osteogenesis

## Abstract

Although the roles of proteoglycans (PGs) have been well documented in the development and homeostasis of the temporomandibular joint (TMJ), how the glycosaminoglycan (GAG) chains of PGs contribute to TMJ chondrogenesis and osteogenesis still requires explication. In this study, we found that FAM20B, a hexokinase essential for attaching GAG chains to the core proteins of PGs, was robustly activated in the condylar mesenchyme during TMJ development. The inactivation of *Fam20b* in craniofacial neural crest cells (CNCCs) dramatically reduced the synthesis and accumulation of GAG chains rather than core proteins in the condylar cartilage, which resulted in a hypoplastic condylar cartilage by severely promoting chondrocyte hypertrophy and perichondral ossification. In the condyles of *Wnt1-Cre;Fam20b^f/f^* mouse embryos, enlarged *Ihh-* and COL10-expressing domains indicated premature hypertrophy resulting from an attenuated IHH-PTHRP negative feedback in condylar chondrocytes, while increased osteogenic markers, canonical Wnt activity, and type-H angiogenesis verified the enhanced osteogenesis in the perichondrium. Further ex vivo investigations revealed that the loss of *Fam20b* decreased the domain area but increased the activity of HH signaling in the embryonic condylar mesenchyme. Moreover, the abrogation of GAG chains in heparan sulfate and chondroitin sulfate proteoglycans led to a rapid up- and then downregulation of HH signaling in condylar chondrocytes, implicating a “slow-release” manner of growth factors controlled by GAG chains. Overall, this study revealed a comprehensive role of the FAM20B-catalyzed GAG chain synthesis in the chondrogenic and osteogenic differentiation of the embryonic TMJ condyle.

## 1. Introduction

Proteoglycans (PGs) are composed of a core protein and one or more glycosaminoglycan (GAG) chains covalently linked to the hydroxyl group of threonine or serine in the core protein. According to the alternative disaccharide units in GAG chains, PGs are categorized into heparan sulfate proteoglycans (HSPGs), chondroitin sulfate proteoglycans (CSPGs), keratan sulfate proteoglycans (KSPGs), and dermatan sulfate proteoglycans (DSPGs) [1,2]. It has been reported that HSPGs and CSPGs play essential roles in chondrogenesis and osteogenesis [3,4,5]. The deletion or mutations of the genes encoding the core proteins of HSPGs or CSPGs, such as *Acan*, *Dcn*, *Hspg2*, *Bgn*, and *Fmod*, could impair skeletogenesis severely [6,7,8,9,10]. Additionally, numerous genetic analyses also revealed a wide spectrum of skeletal disorders resulting from the defects in the GAG chains of PGs [11,12,13,14,15]. Actually, there is accumulated evidence suggesting the role of the GAG chains of PGs in skeletogenesis by controlling the gradients of growth factor and the signaling activity [15,16].

The family with sequence similarity 20 member b (FAM20B) is a newly identified xylose kinase phosphorylating the initial xylose at the tetrasaccharide linkage of GAG chains, which is necessary to attach GAG chains to the core proteins of HSPGs, CSPGs, and DSPGs [17,18,19]. Deficiency of FAM20B destabilizes the tetrasaccharide linkage, which prematurely terminates the synthesis and elongation of GAG chains or detaches GAG chains from the core proteins of HSPGs, CSPGs, and DSPGs [18]. FAM20B-catalyzed GAG chain synthesis has been reported to contribute to skeletogenesis. *Fam20b* deficiency in zebrafish severely impaired bone and cartilage formation [20]. The inactivation of *Fam20b* in the murine skeleton by *Col1-cre* caused a malformed annulus fibrosus in the spine and intervertebral disc by suppressing TGF-β signaling [21], and deleting *Fam20b* in the popliteal cartilage of the mouse knee joint resulted in a chondrosarcoma by enhancing multiple signaling pathways [22].

Skeletogenesis is accomplished by endochondral ossification and intramembranous ossification [23]. The somite-derived axial skeleton and lateral plate mesoderm-derived appendage skeleton are predominantly endochondral ossification, in which the mesenchymal cells first condensed and differentiated into chondrocytes to form a cartilage template and then were replaced by osteoblasts. In contrast, craniofacial skeletogenesis is accomplished mainly by intramembranous ossification, in which the cranial neural crest-derived mesenchymal cells (CNCCs) directly condensed and differentiate into osteoblasts [24,25]. However, the CNCC-derived TMJ condylar mesenchyme condensed in developing mandibular bone first differentiated into condylar cartilage and then underwent endochondral ossification to elongate the mandibular ramus [26,27]. In contrast to the chondrosarcoma in the mesoderm-derived knee cartilage, a hypoplastic condylar cartilage was previously reported when *Fam20b* was inactivated in mouse CNCCs [28], implying an opposite role of *Fam20b* in the CNCC-related chondrogenesis and osteogenesis. In this study, to explore the role of FAM20B-catalyzed GAG chain synthesis in condylar chondrogenesis and osteogenesis, we explored the TMJ development of *Wnt1-Cre;Fam20b^f/f^* mice.

## 2. Results

### 2.1. Fam20b Expression in the Embryonic Condyle Is Essential for GAG Chain Synthesis

**Figure 1:**
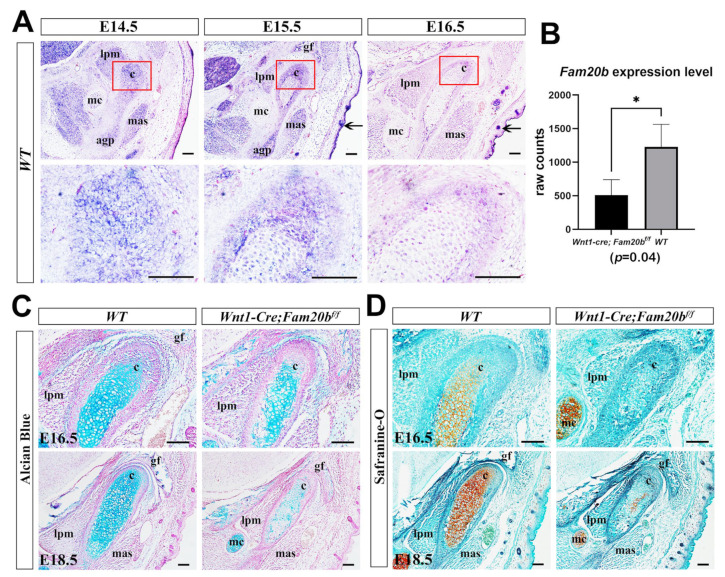
*Fam20b* expression pattern and reduced GAG chains in *Wnt1-Cre;Fam20b^f/f^* TMJ. (**A**) In situ hybridization of *Fam20b* in E14.5, E15.5, and E16.5 TMJ of WT mice. The lower panel shows the magnified views of the red boxes in the corresponding upper panel. Arrows indicate the hair follicles and epidermis. (**B**) Boxplots depicting *Fam20b* transcription in E16.5 WT and *Wnt1-Cre;Fam20b^f/f^* mice (1227 ± 338 vs. 510 ± 229, *p =* 0.04, n = 3). * *p* < 0.05. (**C**,**D**) Alcian blue and Safranin-O fast green staining of E16.5 and E18.5 WT and *Wnt1-Cre;Fam20b^f/f^* TMJ. (c, condyle; mc, Meckel’s cartilage; mas, masseter muscle; lpm, lateral pterygoid muscle; gf, glenoid fossa; agp, angular process; scale bars, 100 μm.)

We first examined the expression pattern of *Fam20b* during TMJ development by in situ hybridization. At E14.5 and E15.5, *Fam20b* was robustly transcribed in the myofibers of craniofacial muscles, Meckel’s cartilage, and the condensed condylar mesenchyme or the differentiating condylar chondrocytes and perichondrium (Figure 1A). At E16.5, *Fam20b* transcription faded obviously in the muscles, Meckel’s cartilages, and the condylar perichondrium and chondrocytes but was still intensive in hair follicles and the epidermis (Figure 1A). To explore the role of *Fam20b* in TMJ development, we generated *Wnt1-Cre;Fam20b^f/f^* mice in which *Fam20b* transcription was abrogated in the CNCCs. Bulk RNA-Seq verified the significantly reduced *Fam20b* transcription in the *Wnt1-Cre;Fam20b^f/f^* condyle (Figure 1B). Histologically, both Alcian blue and Safranin-O staining were dramatically decreased in E16.5 and E18.5 of the *Wnt1-Cre;Fam20b^f/f^* condylar cartilages (Figure 1C,D), implicating that *Fam20b* inactivation reduced the content of GAG chains in the condylar ECM.

### 2.2. The Inactivation of Fam20b in the CNCCs Caused Condylar Hypoplasia by Altering Chondrogenic and Osteogenic Differentiation and Proliferation

**Figure 2:**
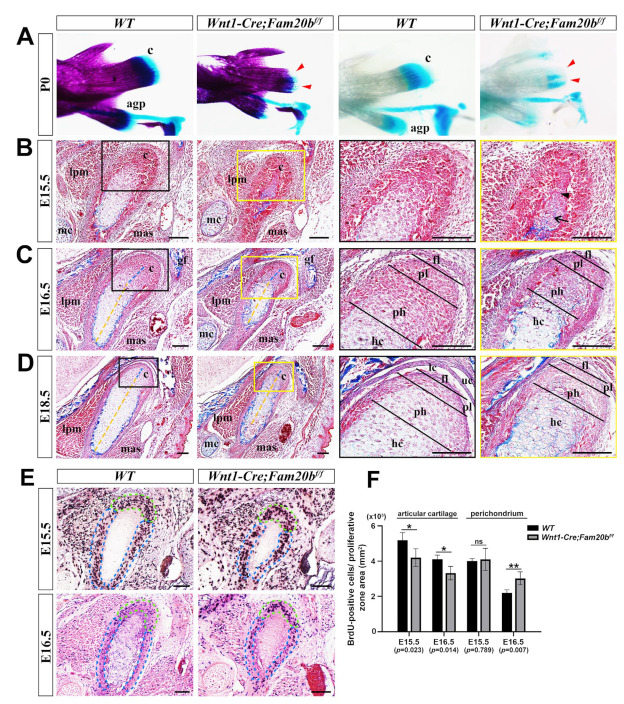
The histological assay of the embryonic *Wnt1-Cre;Fam20b^f/f^* TMJ. (**A**) Whole-mount bone and cartilage staining of P0 WT and *Wnt1-Cre;Fam20b^f/f^* condyles. The red arrowheads indicate the condylar cartilage in the *Wnt1-Cre;Fam20b^f/f^* mandible. (**B**–**D**) Masson staining of the E15.5 (**B**), E16.5 (**C**), and E18.5 (**D**) WT and *Wnt1-Cre;Fam20b^f/f^* TMJ. The black and yellow boxes in the left panels are amplified, respectively, into the corresponding black and yellow boxed images in the right panels. The black arrow points to the hypertrophic chondrocytes, while the black arrowhead points to pre-hypertrophic or proliferating chondrocytes in (**B**). The dashed yellow lines delineate the length of the hypertrophic zone, while the dashed blue lines delineate the pre-hypertrophic zone in (**C**,**D**). (**E**) BrdU labeling shows the proliferating cells in E15.5 and E16.5 WT and *Wnt1-Cre;Fam20b^f/f^* condylar processes. The dashed green lines delineate the articular cartilage, while the dashed blue lines delineate the perichondrium. (**F**) Statistical assay of the density of BrdU-positive cells in (**E**). The densities of proliferating cells are represented as mean ± SD: articular (E15.5: 5193 ± 425 cells/mm^2^ vs. 4199 ± 503 cells/mm^2^, *p* = 0.023, n = 3; E16.5, 4107 ± 234 cells/mm^2^ vs. 3325 ± 388 cells/mm^2^, *p* = 0.014, n = 3) and perichondrium (E15.5, 4104 ± 136 cells/mm^2^ vs. 4104 ± 629 cells/mm^2^, *p* = 0.789, n = 3; E16.5, 2200 ± 171 cells/mm^2^ vs. 3025 ± 371 cells/mm^2^, *p* = 0.007, n = 3); ** *p* < 0.01. * *p* < 0.05. (c, condyle; mc, Meckel’s cartilage; mas, masseter muscle; lpm, lateral pterygoid muscle; gf, glenoid fossa; agp, angular process; uc, upper joint cavity; lc, lower joint cavity; fl, fibrous cell layer; pl, proliferative cell layer; ph, pre-hypertrophic chondrocytes; hc, hypertrophic chondrocytes; scale bars, 100 μm.)

Compared with WT controls, the P0 *Wnt1-Cre;Fam20b^f/f^* condylar cartilage displayed a remarkable hypoplasia in whole-mount bone and cartilage staining (Figure 2A). Although the perichondrium, the presumptive disc, and the emerging bone collar were discernible, the E15.5 *Wnt1-Cre;Fam20b^f/f^* condyle contained fewer and disorganized pre-hypertrophic and hypertrophic chondrocytes, instead of the distinguishable proliferating, pre-hypertrophic, and hypertrophic layers in the WT condyle (Figure 2B). When the E16.5 *Wnt1-Cre;Fam20b^f/f^* condyle displayed discernible superficial, proliferating, and pre-hypertrophic layers, the ratio of the proliferating and pre-hypertrophic zone to total condylar cartilage was obviously decreased, while the ratio of the hypertrophic layer was increased (Figure 2C). Worthy of note, the collagen deposition in the E15.5 *Wnt1-Cre;Fam20b^f/f^* condylar perichondrium was also more evident (Figure 2B,C). The increasing hypertrophic zone, the decreasing proliferating and pre-hypertrophic zones, and the enhanced collagen deposition became more noticeable in the E18.5 *Wnt1-Cre;Fam20b^f/f^* condyle than in the WT counterparts (Figure 2D). Consistently, the density of BrdU-positive cells was significantly reduced in the proliferating and articular layers but noticeably increased in the perichondrium of *Wnt1-Cre;Fam20b^f/f^* condyles (Figure 2E,F). Meanwhile the TUNEL assay showed no difference in cell apoptosis between WT and *Wnt1-Cre;Fam20b^f/f^* condyles (Figure S1). Taken together, the chondrogenic and osteogenic differentiation and the proliferation in the TMJ condyle were altered by the loss of GAG chains.

### 2.3. Premature Hypertrophy in Wnt1-Cre;Fam20b^f/f^ Condylar Chondrocytes

**Figure 3:**
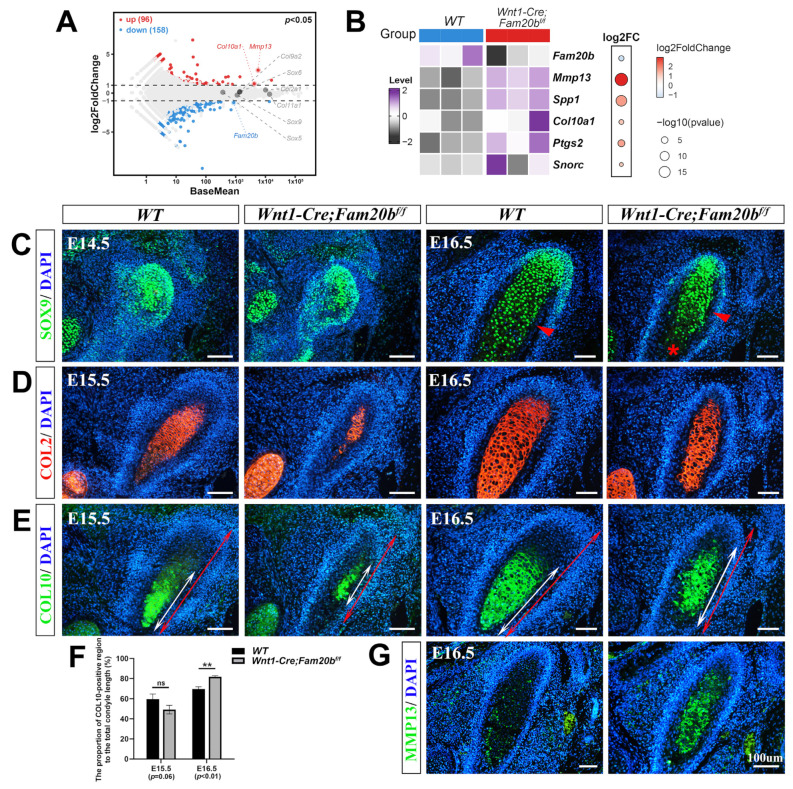
The premature differentiation of condylar chondrocytes in *Wnt1-Cre;Fam20b^f/f^* TMJ. (**A**) Volcano plot showing the DEGs in the E16.5 *Wnt1-Cre;Fam20b^f/f^* vs. WT condyles. (**B**) Heat map showing the chondrogenic differentiation- and maturation-related genes in the DEGs. The color and size of dots in the right box represent gene expression abundance and *p*-value, respectively. (**C**) Immunofluorescence staining of SOX9 in the E14.5 and E16.5 WT and *Wnt1-Cre;Fam20b^f/f^* TMJ. The red arrowheads point to the bone collar, and the asterisk points to premature hypertrophic chondrocytes. (**D**,**E**) Immunofluorescence staining of COL2 (**D**) and COL10 (**E**) in E15.5 and E16.5 WT and *Wnt1-Cre;Fam20b^f/f^* mice. The white double-head arrows delineate COL10-positive portions, and the red double-head arrows delineate the total condyle length. (**F**) Statistical analysis shows the proportions of COL10-positive area to the total length of condylar cartilage in (**E**). Data are represented as mean ± SD: E15.5, 59.58 ± 4.3% vs. 49.26 ± 3.51%, *p* = 0.06, n = 3, and E16.5, 69.74 ± 2.33% vs. 81.77 ± 1.21%, *p* < 0.01, n = 3. (**G**) Immunofluorescence staining of MMP13 in WT and *Wnt1-Cre;Fam20b^f/f^* TMJ. (** *p* < 0.01; scale bars, 100 μm.)

With the significance threshold of |log2FC| > 1 and *p* < 0.05, bulk RNA-Seq identified 254 differential expression genes (DEGs) in which 96 were upregulated and 158 downregulated significantly in the *Wnt1-Cre;Fam20b^f/f^* condyles compared with the WT counterparts (Figure 3A). Among the DEGs, the increased expression of *Mmp13*, *Spp1*, *Col10a1*, *Ptgs2*, and *Snorc* in the *Wnt1-Cre;Fam20b^f/f^* condyle suggests an enhanced differentiation and ossification (Figure 3B). The SOX9 immunostaining spreading throughout the E14.5 condylar mesenchyme suggests the unaltered chondro-osteogenic fate of the *Wnt1-Cre;Fam20b^f/f^* TMJ progenitors; however, the reduced SOX9 staining in the E16.5 *Wnt1-Cre;Fam20b^f/f^* hypertrophic zone implicates the fewer proliferating chondrocytes resulting from an enhanced chondrogenic differentiation (Figure 3C). Another marker of proliferating chondrocytes, the COL2 staining was obviously reduced in the E15.5 *Wnt1-Cre;Fam20b^f/f^* condyle but comparable with that in the WT condyle at E16.5 (Figure 3D). The hallmark of hypertrophic chondrocytes, COL10, located in the relatively lower portion in both the E15.5 and E16.5 WT condyles, was detected in an upper portion in the E15.5 *Wnt1-Cre;Fam20b^f/f^* condyles (Figure 3E) and extended into almost the entire E16.5 *Wnt1-Cre;Fam20b^f/f^* condyles (Figure 3F). More convincingly, both the rapid extension and overlapping of the COL2 and COL10 staining in the *Wnt1-Cre;Fam20b^f/f^* condyles suggest an accelerated differentiation of condylar mesenchymal progenitors into proliferating chondrocytes and a premature hypertrophy of condylar proliferating chondrocytes in the *Wnt1-Cre;Fam20b^f/f^* condyles, respectively. Additionally, the area of MMP13 staining was also remarkably enlarged in the *Wnt1-Cre;Fam20b^f/f^* condyle (Figure 3G), implicating a much more robust ECM remodeling that was essential for the hypertrophy and endochondral ossification of the condylar chondrocytes. Taken together, all these data indicate that, in the *Wnt1-Cre;Fam20b^f/f^* condyle, the loss of GAG chains leads to the premature differentiation of chondrocytes.

### 2.4. The Loss of Fam20b Promoted Osteogenic Differentiation in the Condylar Perichondrium

**Figure 4:**
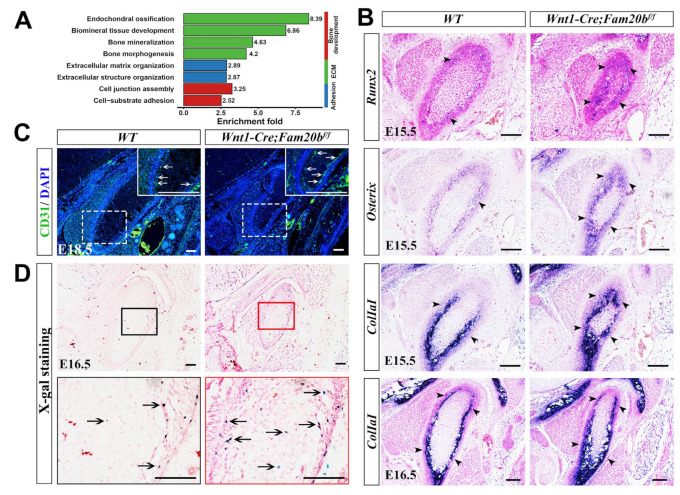
The perichondrial osteogenesis in *Wnt1-Cre;Fam20b^f/f^* TMJ. (**A**) GO enrichment indicates that the DEGs were mainly involved in bone-, ECM-, and adhesion-related biological processes. (**B**) In situ hybridization of *Runx2*, *Osterix*, and *Col1a1* in E15.5 and E16.5 WT and *Wnt1-Cre;Fam20b^f/f^* TMJ. The black arrowheads indicate positive cells in the WT and *Wnt1-Cre;Fam20b^f/f^* condyles. (**C**) Immunofluorescence staining of CD31 in E18.5 *WT* and *Wnt1-Cre;Fam20b^f/f^* TMJ. The areas in the white dashed boxes are amplified into the boxes with solid white lines, in which the white arrows indicate CD31-positive cells. (**D**) X-gal/LacZ staining in the E16.5 *WT* (left panel) and *Wnt1-Cre;Fam20b^f/f^;BATgal* TMJ (right panel). The areas in the black and red boxes are amplified into the black and red boxed images, respectively. The black arrows indicate LacZ-positive cells. Scale bars, 100 μm.

Since MMP13 also acts as an indicator of endochondral ossification [29,30], we further explored the osteogenesis in the *Wnt1-Cre;Fam20b^f/f^* condyle. Bulk RNA-Seq analysis revealed that the DEGs were mainly enriched in bone-, ECM-, and adhesion-related biological processes (Figure 4A). In situ hybridization verified that the transcription and expressing-domain of *Runx2*, *Osterix*, and *Col1a1* were all significantly upregulated and enlarged in the E15.5 and E16.5 *Wnt1-Cre;Fam20b^f/f^* perichondrium (Figure 4B), which coincided with the enhanced collagen deposition (Figure 2C,D). Additionally, CD31, an endothelial marker of type H vessels, which is coupled with de novo bone formation, was much more intensive in the *Wnt1-Cre;Fam20b^f/f^* TMJ perichondrium (Figure 4C). Combined with the more LacZ-positive cells in the condylar perichondrium of E16.5 *Wnt1-Cre;Fam20b^f/f^;BATgal* mice (Figure 4D), representing an upregulated canonical Wnt activity in the condylar perichondrium, all these consequences indicate enhanced osteogenesis in the condylar perichondrium.

### 2.5. Upregulated Ihh Transcription and HH Signaling Activity in the Wnt1-Cre;Fam20b^f/f^ Condyle

**Figure 5:**
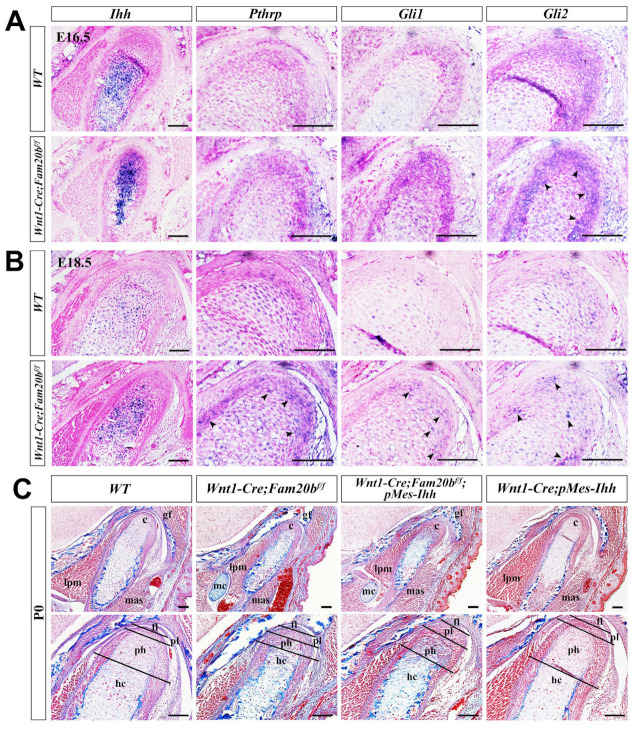
The regulation of GAG chains on IHH signaling in TMJ condylar cartilages. (**A**,**B**) In situ hybridization of *Ihh*, *Pthrp*, *Gli1*, and *Gli2* expression pattern in E16.5 (**A**) and E18.5 (**B**) WT and *Wnt1-Cre;Fam20b^f/f^* condylar cartilage. The black arrowheads indicate the upregulated positive signals in *Wnt1-Cre;Fam20b^f/f^* condyles. (**C**) Masson staining of the P0 WT, *Wnt1-Cre;Fam20b^f/f^*, *Wnt1-Cre;Fam20b^f/f^;pMes-Ihh*, and *Wnt1-Cre;pMes-Ihh* condyles. (c, condyle; mc, Meckel’s cartilage; mas, masseter muscle; lpm, lateral pterygoid muscle; gf, glenoid fossa; fl, fibrous cell layer; pl, proliferative cell layer; ph, pre-hypertrophic chondrocytes; hc, hypertrophic chondrocytes; scale bars, 100 μm.)

Indian Hedgehog (IHH) and Hedgehog (HH) signaling play critical roles in chondrogenic differentiation and endochondral ossification [31]. In situ hybridization showed a remarkably enhanced *Ihh* transcription almost throughout the E16.5 *Wnt1-Cre;Fam20b^f/f^* condylar cartilage (Figure 5A), which faded but was still more robust in the E18.5 *Wnt1-Cre;Fam20b^f/f^* condylar cartilage than in the WT condyle (Figure 5B). However, only *Gli2* expression became more robust in the E16.5 *Wnt1-Cre;Fam20b^f/f^* condylar cartilages, while the *Pthrp* and *Gli1* expression seemed no different from that in the WT controls (Figure 5A), suggesting an upregulated IHH-GLI2 signaling transduction in the *Wnt1-Cre;Fam20b^f/f^* condyle. At E18.5, the transcriptions of *Pthrp*, *Gli1*, and *Gli2* were all elevated compared with those in the WT controls (Figure 5B), indicating a more robust IHH signaling in the *Wnt1-Cre;Fam20b^f/f^* condylar cartilage than that in E16.5. Then, we further assessed the role of GAG chains in regulating HH signaling activity by activating a conditional *Ihh* transgene in *Wnt1-cre* mice (*Wnt1-cre;pMes-Ihh*). Notably, the *Wnt1-Cre;pMes-Ihh* mouse embryos showed a comparable TMJ phenotype to the WT control, while the *Wnt1-Cre;Fam20b^f/f^;pMes-Ihh* mice exhibited an expanded hypertrophic zone and enhanced ossification in the TMJ condyle, which phenocopied *Wnt1-Cre;Fam20b^f/f^* mice (Figure 5C), further verifying that FAM20B-catalyzed GAG chains are indispensable in IHH diffusion and HH signaling activity.

### 2.6. The Loss of Fam20b Impaired the Diffusion but Accelerated the Release of Sonic Hedgehog (SHH) Without Altering the ECM Content in the Condylar Cartilage

**Figure 6:**
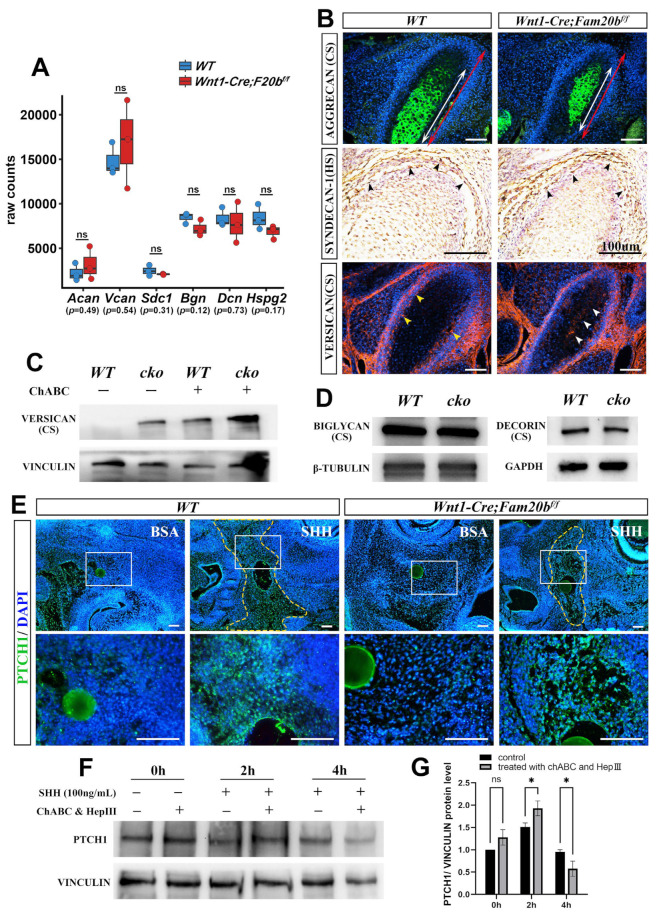
The PG contents, SHH diffusion, and dynamic activity of HH signaling in the absence of GAG chains. (**A**) Boxplots depicting *Aggrecan* (*Acan*), *Versican* (*Vcan*), *Syndecan-1* (*Sdc1*), *Biglycan* (*Bgn*), *Decorin* (*Dcn*), and *Perlecan* (*Hspg2*) transcription in E16.5 WT and *Wnt1-Cre;Fam20b^f/f^* mice. The blue rectangles represent WT, and the red rectangle represents *Wnt1-Cre;Fam20b^f/f^* TMJ. (**B**) Immunostaining of AGGRECAN, SYNDECAN-1, and VERSICAN in E16.5 WT and *Wnt1-Cre;Fam20b^f/f^* condyles. The white double-head arrows delineate AGGRECAN-positive portions, while the red double-head arrows delineate the total condyle length. The black arrowheads indicate SYNDECAN-1-positive cells. The yellow arrowheads indicate VERSICAN in the perichondrium of WT condyles, while the white arrowheads indicate VERSICAN in the central region in *Wnt1-Cre;Fam20b^f/f^* condyles. (**C**) Western blots for VERSICAN in WT and *Wnt1-Cre;Fam20b^f/f^* condyles. GAG chains were eliminated by chondroitinase ABC to allow full migration into SDS-PAGE gel. (**D**) Western blots for BIGLYCAN and DECORIN in WT and *Wnt1-Cre;Fam20b^f/f^* condyles. (**E**) Immunostaining of PTCH1 in E13.5 WT and *Wnt1-Cre;Fam20b^f/f^* TMJ explants cultured with BSA or SHH beads (n = 4). The yellow dashed line shows that the PTCH1 domain in WT was significantly larger than that in *Wnt1-Cre;Fam20b^f/f^* mice after 16 h culture with SHH-soaked beads. The lower panel shows the magnified views of the white boxes in the corresponding upper panel. (**F**,**G**) Western blot analysis (**F**) and quantitative data (**G**) of PTCH1 contents in control and chondroitinase ABC- and heparinase III-treated condylar chondrocytes after 0 h, 2 h, and 4 h incubation with SHH, respectively. (ChABC, chondroitinase ABC; HepIII, heparinase III; * *p* < 0.05; scale bars, 100 μm.)

To reveal how GAG chains regulate the activity of HH signaling in vivo, we first conducted quantitative analysis and immunostaining of CSPGs or HSPGs in *Wnt1-Cre;Fam20b^f/f^* condylar cartilage. Bulk RNA-Seq revealed that the transcription of *Aggrecan* (*Acan*), *Versican* (*Vcan*), *Syndecan-1* (*Sdc1*), *Biglycan* (*Bgn*), *Decorin* (*Dcn*), and *Perlecan* (*Hspg2*) showed no differences between the WT and *Wnt1-Cre;Fam20b^f/f^* condyles (Figure 6A). The immunostaining of AGGRECAN, the richest CSPG in the cartilage matrix, and SYNDECAN-1, a HSPG in the cell surface, showed comparable intensity and distribution in the *Wnt1-Cre;Fam20b^f/f^* condylar cartilages and WT controls (Figure 6B). VERSICAN, a large CSPG, displayed an extra domain in the central cartilage but a reduced intensity in the perichondrium of the *Wnt1-Cre;Fam20b^f/f^* condyle (Figure 6B). After eliminating the GAG chains of VERSICAN by chondroitinase ABC treatment [32], the Western blots exhibited little difference between the WT and *Wnt1-Cre;Fam20b^f/f^* condyles (Figure 6C; Figure S2). Similarly, Western blots also displayed little difference in BIGLYCAN and DECORIN between the WT and *Wnt1-Cre;Fam20b^f/f^* condylar cartilages (Figure 6D; Figure S2). All these consequences confirmed the little impacts on the expression of the core proteins of HSPG and CSPG in *Wnt1-Cre;Fam20b^f/f^* condyles.

Then, we explored the role of FAM20B-catalyzed GAG chains in growth factor diffusion by implanting SHH-soaked beads into the condensing E13.5 condylar mesenchyme. We found that the SHH-soaked beads activated a significantly larger PTCH1-positive domain in the WT control than that in the *Wnt1-Cre;Fam20b^f/f^* counterpart (Figure 6E; Figure S2). Furthermore, ablating GAG chains from the E16.5 WT condylar chondrocytes by chondroitinase ABC and heparinase III led to a faster and higher PTCH1 intensity in 2 h, which plummeted to a lower intensity in 4 h after SHH supplement (Figure 6F,G). Both the milder activity fluctuation in the WT condylar chondrocytes without chondroitinase ABC treatment and the larger HH signaling domain in the WT condylar mesenchyme with SHH supplement suggest that the FAM20B-catalyzed GAG chain synthesis not only facilitates the diffusion of IHH but also maintains the moderate activity of HH signaling by controlling IHH release in the condylar cartilage.

## 3. Discussion

The roles of PGs in chondrogenesis and osteogenesis have been well documented in the mesoderm-derived skeleton [33,34]. However, there are only a few studies concerning the role of PGs in CNCC-related chondrogenesis and osteogenesis [27,28,35]. In the present study, we demonstrated that deleting *Fam20b* in CNCCs disabled the GAG chain synthesis of PGs, which resulted in a premature differentiation of condylar chondrocytes and an enhanced perichondrial osteogenesis. In vivo explorations revealed an upregulated *Ihh* expression and enhanced HH signaling activity due to the loss of GAG chains. Further ex vivo investigation suggested that the upregulated activity of Hh signaling in the absence of GAG chains resulted from a rapid release of ligands at the expense of activity persistence. Moreover, the reduced SHH diffusion in the *Wnt1-Cre;Fam20b^f/f^* condylar mesenchyme suggested that the GAG chains of PGs facilitated IHH diffusion in the condylar cartilage.

During long bone development, *Ihh* was expressed in the pre-hypertrophic and hypertrophic chondrocytes, which, in turn, activated *Pthrp* expression in the proliferating chondrocytes [36,37]. PTHRP maintained the proliferation of chondrocytes but suppressed *Ihh* expression and chondrocyte hypertrophy, which forms a negative feedback loop with IHH during cartilage development [38,39]. The increasing proliferation enlarged the distance between PTHRP-secreting and proliferating chondrocytes, which released *Ihh* expression from PTHRP suppression. The *Ihh* activation was sufficient to promote chondrogenic hypertrophy in both long bone and TMJ condylar chondrocytes [31,38]. Thus, we speculated that, in the *Wnt1-Cre;Fam20b^f/f^* condylar cartilage, the limited diffusion of *Ihh* delayed the activation of *Pthrp* in the superficial layer, leading to the reduced proliferation and the thinner proliferating zone. In turn, without the suppression by PTHRP, IHH promoted a premature differentiation and maturation of the condylar chondrocytes. Therefore, the hypoplastic *Wnt1-Cre;Fam20b^f/f^* condylar cartilages exhibited the much thinner proliferating zones, the much more robust *Ihh* transcription, and the obviously increased ratio of the hypertrophic zone.

Along with the premature differentiation of condylar chondrocytes, the osteogenesis in the *Wnt1-Cre;Fam20b^f/f^* condylar perichondrium was also enhanced, which coincided with the *Fam20b^b1127^* (null mutation) zebrafish and *pug* (*Xylt1* missense mutation) mice [20,40]. Recent studies indicate that, in postnatal long bone, IHH produced by the pre-hypertrophic and hypertrophic chondrocytes in growth plates not only promotes trabecular bone formation but also enhances the perichondrial osteoblasts to form cortical bone [41,42]. Interestingly, the elevated IHH signaling in the perichondrium also enhanced the osteogenesis for the bone cortex by upregulating canonical Wnt signaling [43,44]. Consistently, our study displayed elevated HH and canonical Wnt signaling activity in the *Wnt1-Cre;Fam20b^f/f^* condylar perichondrium. Similarly, previous studies have indicated that the loss of *Fam20b* in epithelial cells or the cartilage of the knee joint resulted in the hyperactivation of FGF, HH, Erk, BMP, and Wnt signaling [22,45]. The latest study also showed increasing BMP-Smad signaling during the endochondral ossification of *Fam20b*-mutant zebrafish, while inhibition of BMP-Smad signaling rescued the premature cartilage [46]. Combined with all these consequences, the loss of *Fam20b* that disabled GAG chain synthesis was suggested to discriminately upregulate the intensity of multiple signaling. Furthermore, when GAG chains were absent, the ex vivo expansion and dramatic fluctuation of HH signaling suggested that FAM20B-catalyzed GAG chain synthesis was required to finely tune the binding and releasing of ligands, which maintained the moderate intensity and range of signaling.

Of note, in our study, both *Fam20b* inactivation and enzyme treatment abrogated GAG chains in HSPGs and CSPGs indiscriminately; therefore, the decreased diffusion of SHH and the increased HH signaling activity were the net outcome of the GAG chain deficiency in HSPGs and CSPGs. Previous studies revealed that HSPGs restricted IHH diffusion by sequestering IHH on the cell surface, through which the local IHH concentration was increased [13,15], whereas the deficient sulfate modification in the GAG chains of CSPGs disclosed that the CSPGs facilitated IHH diffusion as a widely distributed conductor [16]. Of note, besides the comparable amounts of BIGLYCAN and DECORIN in *Wnt1-Cre;Fam20b^f/f^* and WT condyles, Western blots also showed the similar amounts of VERSICAN after chondroitinase ABC treatment. The absence of a VERSICAN band from the WT samples without chondroitinase ABC treatment most likely resulted from the failed entry of VERSICAN into the PAGE gel or the recognizing sites of the antibody being covered by the attached GAG chains. Combined with all these results, it is suggested that the amounts of the core proteins of CSPG and HSPG were changed little by the deficiency of *Fam20b*, indicating that it was the GAG chains that regulated signaling intensity. The phenotype similarities between *Wnt1-Cre;Fam20b^f/f^;pMes-Ihh* and *Wnt1-Cre;Fam20b^f/f^* mice, as well as between *Wnt1-Cre;pMes-Ihh* and WT mice, further verify the indispensability of GAG chains in the buffering on signaling intensity.

Although FAM20B-catalyzed GAG chain synthesis maintained the moderate activity and distribution of HH signaling during condylar chondrogenesis and osteogenesis by finely tuning the releasing and diffusion of IHH, further exploration is still required to clarify whether the enhanced osteogenesis was achieved via the substitution of hypertrophic chondrocytes by osteoblasts as in canonical endochondral ossification [23,47] or via the direct transformation of chondrocytes into osteocytes [48,49,50,51,52]. Furthermore, the detachment of GAG chains from PGs is a characteristic of aging, which is regarded as one of the main causes of osteoarthritis [53,54]. There is an opinion attributing the formation of osteophytes in osteoarthritis to the pathological transformation of hypertrophic chondrocytes into osteocytes [55,56]. As aforementioned, *Pthrp* transcription was upregulated in the *Wnt1-Cre;Fam20b^f/f^* condylar cartilages. A recent review summarized that PTHRP not only contributed to in vivo angiogenesis during endochondral ossification but also downregulated the expression of *Sclerostin* and *Dickkopf-1*, the inhibitors of Wnt signaling [57]. These findings are consistent with the increased angiogenic markers and Wnt signaling in the enhanced perichondrial osteogenesis of the *Wnt1-Cre;Fam20b^f/f^* condyles. Therefore, such a proposed correlation between deficiency in GAG chains and chondrocyte transformation or perichondrial osteogenesis implies a potential role of GAG chains in osteoarthritis, which is worthy of further exploration.

## 4. Materials and Methods

### 4.1. Mouse Lines and Ethical Statement

All *Wnt1-Cre*, *Fam20b^f/f^*, *BATgal*, and *pMes-Ihh* mice were maintained and expanded in the Specific Pathogen-Free System of the Institute of Genome Engineered Animal Models for Human Diseases at the Dalian Medical University. The genotyping protocols have been described previously [58,59]. All the animal experiments were approved by the Animal Care and Use Committee at the Dalian Medical University (Protocol No. AEE17038).

### 4.2. Histology, Immunohistochemistry, Immunofluorescence Staining, and In Situ Hybridization

The heads dissected from timed mouse embryos were fixed with 4% paraformaldehyde overnight, embedded with paraffin after gradient alcohol dehydration, and sectioned at the thickness of 10 μm. The TMJ histology was examined by Masson staining (Biebrich scarlet-acid fuchsin solution and Aniline Blue), Alcian blue staining, and Safranin O/Fast Green staining, while whole-mount bone and cartilage staining was achieved by Alizarin Red S (0.1% in ethanol) and Alcian Blue (0.3% in ethanol). For immunofluorescence staining, the antibodies against SOX9 (1:1000 dilution, ab185966, Abcam, Cambridge, MA, USA), COL2 (1:800 dilution, BA0533, BOSTER, Pleasanton, CA, USA), COL10 (1:1000 dilution, BA2023, BOSTER), AGGRECAN (1:1500 dilution, 13880-1-AP, Proteintech, Wuhan, China), VERSICAN (1:1000 dilution, ab269270, Abcam), MMP13 (1:600 dilution, 18165-1-AP, Proteintech), CD31 (1:300 dilution, ab28364, Abcam), and PTCH1 (1:100, YT3598, Immunoway, Plano, TX, USA) were used as primary antibodies, while goat anti-rabbit IgG H&L (Alexa Fluor^®^ 647 for red fluorescence) (1:400 dilution, ab150079, Abcam) and goat anti-rabbit IgG H&L (Alexa Fluor^®^ 488 for green fluorescence) (1:400 dilution, ab150077, Abcam) were used as secondary antibodies. A solution of 4′,6-diamidino-2-phenylindole dihydrochloride (DAPI) was applied for conterstaining (HY-D0814, MedChemExpress LLC, Shanghai, China). For immunohistochemical staining, the primary antibody against SYNDECAN-1 (1:2500 dilution, ab128936, Abcam) was used. The MaxVision TM HRP Polymer anti-Mouse/Rabbit IHC Kit (No. KIT5020, Maixin Ltd., Fuzhou, China) was used as secondary antibody, the 3,3′-diaminobenzidine (No. DAB-0031, Maixin Ltd.) for yellow/brown color development and the solution of hematoxylin for counterstaining. For in situ hybridization, sections were incubated with digoxygenin-labeled (No. 11277073910, Roche, Basel, Switzerland) antisense RNA probes to *Fam20b*, *Ihh*, *Pthrp*, *Gli1*, *Gli2*, *Runx2*, *Osterix*, and *Col1a1* as described previously [35,60]. The alkaline phosphatase-conjugated antibody against digoxin (No. 11093274910, Roche, Basel, Switzerland) was applied as the secondary antibody for blue/black color development. The sections were counterstained with eosin into red.

### 4.3. Cell Proliferation and Apoptosis Assays

The timed pregnant mice were intraperitoneally injected with BrdU labeling solution (10 mg/mL) at a dose of 10 uL/g body weight half an hour prior to euthanizing. The embryonic heads were fixed with Carnoy fixed solution for 4 h, dehydrated by gradient ethanol, and sectioned at 10 μm. A cell proliferation assay was performed using Detection Kit II (No. 11299964001, Roche). The apoptosis assay was conducted using the In Situ Cell Death Detection Kit, POD (No. 11684817910, Roche).

### 4.4. Western Immunoblotting

The collected condyles were treated in RIPA buffer supplemented with PMSF and chondroitinase ABC (0.5 U/mL, C2905, Sigma, St. Louis, MO, USA) and loaded into 7.5% or 10% SDS-PAGE gels. PVDF membranes (No. 10600023, Cytiva, Washington, DC, USA) were incubated with antibodies against BIGLYCAN (1:1000 dilution, 16409-1-AP, Proteintech), DECORIN (1:1000 dilution, 14667-1-AP, Proteintech), VERSICAN (1:1000 dilution, ab269270, Abcam), PTCH1 (1:500 dilution, YT3598, Immunoway), GAPDH (1:10,000 dilution, ET1601-4, Huabio, Hangzhou, China), VINCULIN (1:10,000, 66305-1-Ig, Proteintech), and β-TUBULIN (1:8000, 10094-1-AP, Proteintech). Goat anti-rabbit IgG (1:10,000 dilution, AS014, ABclonal, Wuhan, China) and goat anti-mouse IgG (1:10,000 dilution, AS003, ABclonal) were applied as secondary antibodies.

### 4.5. Cell Culture, In Vitro Organ Culture, and Bead Implantation

The E16.5 WT condylar chondrocytes were treated with chondroitinase ABC (0.05 U/mL) and heparinase III (0.05 U/mL, H8891, Sigma) for 72–96 h. Then, recombinant human Sonic Hedgehog (SHH) protein (100 ng/mL, HZ-1306, Proteintech) was supplemented for a 2 to 4 h culture prior to protein extraction for the Western blot. SHH (0.5 μg/μL)-soaked agarose beads (1537302, Bio-Rad, Hercules, CA, USA) were grafted in E13.5 mouse mandibles for a 16 h culture. The explants were fixed in 4% paraformaldehyde for immunofluorescence staining.

### 4.6. Bulk RNA-Sequencing

The Illumina platform was utilized for bipartite sequencing, with the total number of bases per sample fluctuating around 6G. Trim_galore was utilized to remove low-quality reads and splice sequences; Histat2 aligned the reference genome to the mouse reference genome mm10; and FeatureCounts counted the number of reads landing on protein-coding genes. To obtain the gene expression profile, the count matrix was transformed into an FPKM matrix, followed by log transformation for the *t*-tests of specific genes. The mean difference in expression of the three replicated sets was used as the foldchange. The RNA-Seq data were deposited in the Gene Expression Omnibus (GEO) database with BioProject number PRJNA1102296, and the acquisition linkage was as follows: https://dataview.ncbi.nlm.nih.gov/object/PRJNA1102296?reviewer=irduarmjb74td17qdo74kn9t2h, accessed on 24 May 2024.

### 4.7. Statistical Analysis

At least three replicates were performed independently for each statistical analysis. Data were determined using a 2-tailed Student’s *t*-test with *p* < 0.05 as statistically significant and represented as mean ± SD. All the statistics were conducted using GraphPad Prism software, version 9.5.1 (GraphPad Software Inc., San Diego, CA, USA).

## Data Availability

All datasets in the present study are available from the corresponding author upon reasonable request.

## Supplementary Materials

The following supporting information can be downloaded at https://www.mdpi.com/article/10.3390/ijms26094033/s1.

## References

[1] Paganini C., Costantini R., Superti-Furga A., Rossi A. (2019). Bone and connective tissue disorders caused by defects in glycosaminoglycan biosynthesis: A panoramic view. FEBS J..

[2] Theocharis A.D., Skandalis S.S., Gialeli C., Karamanos N.K. (2016). Extracellular matrix structure. Adv. Drug Deliv. Rev..

[3] Schwartz N.B., Domowicz M.S. (2022). Roles of Chondroitin Sulfate Proteoglycans as Regulators of Skeletal Development. Front. Cell Dev. Biol..

[4] Jochmann K., Bachvarova V., Vortkamp A. (2014). Heparan sulfate as a regulator of endochondral ossification and osteochondroma development. Matrix Biol..

[5] Karamanos N.K., Piperigkou Z., Theocharis A.D., Watanabe H., Franchi M., Baud S., Brezillon S., Gotte M., Passi A., Vigetti D. (2018). Proteoglycan Chemical Diversity Drives Multifunctional Cell Regulation and Therapeutics. Chem. Rev..

[6] Lauing K.L., Cortes M., Domowicz M.S., Henry J.G., Baria A.T., Schwartz N.B. (2014). Aggrecan is required for growth plate cytoarchitecture and differentiation. Dev. Biol..

[7] Chery D.R., Han B., Zhou Y., Wang C., Adams S.M., Chandrasekaran P., Kwok B., Heo S.J., Enomoto-Iwamoto M., Lu X.L. (2021). Decorin regulates cartilage pericellular matrix micromechanobiology. Matrix Biol..

[8] Han B., Li Q., Wang C., Patel P., Adams S.M., Doyran B., Nia H.T., Oftadeh R., Zhou S., Li C.Y. (2019). Decorin Regulates the Aggrecan Network Integrity and Biomechanical Functions of Cartilage Extracellular Matrix. ACS Nano.

[9] Embree M.C., Kilts T.M., Ono M., Inkson C.A., Syed-Picard F., Karsdal M.A., Oldberg Å., Bi Y., Young M.F. (2010). Biglycan and Fibromodulin Have Essential Roles in Regulating Chondrogenesis and Extracellular Matrix Turnover in Temporomandibular Joint Osteoarthritis. Am. J. Pathol..

[10] Lowe D.A., Lepori-Bui N., Fomin P.V., Sloofman L.G., Zhou X., Farach-Carson M.C., Wang L., Kirn-Safran C.B. (2014). Deficiency in perlecan/HSPG2 during bone development enhances osteogenesis and decreases quality of adult bone in mice. Calcif. Tissue Int..

[11] Mizumoto S., Yamada S. (2021). Congenital Disorders of Deficiency in Glycosaminoglycan Biosynthesis. Front. Genet..

[12] Dubail J., Huber C., Chantepie S., Sonntag S., Tüysüz B., Mihci E., Gordon C.T., Steichen-Gersdorf E., Amiel J., Nur B. (2018). SLC10A7 mutations cause a skeletal dysplasia with amelogenesis imperfecta mediated by GAG biosynthesis defects. Nat. Commun..

[13] Yasuda T., Mundy C., Kinumatsu T., Shibukawa Y., Shibutani T., Grobe K., Minugh-Purvis N., Pacifici M., Koyama E. (2010). Sulfotransferase Ndst1 is Needed for Mandibular and TMJ Development. J. Dent. Res..

[14] Wilson D.G., Phamluong K., Lin W.Y., Barck K., Carano R.A.D., Diehl L., Peterson A.S., Martin F., Solloway M.J. (2012). Chondroitin sulfate synthase 1 (Chsy1) is required for bone development and digit patterning. Dev. Biol..

[15] Koziel L., Kunath M., Kelly O.G., Vortkamp A. (2004). Ext1-Dependent Heparan Sulfate Regulates the Range of Ihh Signaling during Endochondral Ossification. Dev. Cell..

[16] Cortes M., Baria A.T., Schwartz N.B. (2009). Sulfation of chondroitin sulfate proteoglycans is necessary for proper Indian hedgehog signaling in the developing growth plate. Development.

[17] Koike T., Izumikawa T., Tamura J.-I., Kitagawa H. (2009). FAM20B is a kinase that phosphorylates xylose in the glycosaminoglycan–protein linkage region. Biochem. J..

[18] Wen J., Xiao J., Rahdar M., Choudhury B.P., Cui J., Taylor G.S., Esko J.D., Dixon J.E. (2014). Xylose phosphorylation functions as a molecular switch to regulate proteoglycan biosynthesis. Proc. Natl. Acad. Sci. USA.

[19] Zhang H., Zhu Q., Cui J., Wang Y., Chen M.J., Guo X., Tagliabracci V.S., Dixon J.E., Xiao J. (2018). Structure and evolution of the Fam20 kinases. Nat. Commun..

[20] Wilkie A.O.M., Eames B.F., Yan Y.-L., Swartz M.E., Levic D.S., Knapik E.W., Postlethwait J.H., Kimmel C.B. (2011). Mutations in fam20b and xylt1 Reveal That Cartilage Matrix Controls Timing of Endochondral Ossification by Inhibiting Chondrocyte Maturation. PLoS Genet..

[21] Saiyin W., Li L., Zhang H., Lu Y., Qin C. (2019). Inactivation of FAM20B causes cell fate changes in annulus fibrosus of mouse intervertebral disc and disc defects via the alterations of TGF-β and MAPK signaling pathways. Biochim. Biophys. Acta Mol. Basis Dis..

[22] Ma P., Yan W., Tian Y., Wang J., Feng J.Q., Qin C., Cheng Y.S., Wang X. (2016). Inactivation of Fam20B in Joint Cartilage Leads to Chondrosarcoma and Postnatal Ossification Defects. Sci. Rep..

[23] Galea G.L., Zein M.R., Allen S., Francis-West P. (2021). Making and shaping endochondral and intramembranous bones. Dev. Dyn..

[24] Berendsen A.D., Olsen B.R. (2015). Bone development. Bone.

[25] Parada C., Chai Y. (2015). Mandible and Tongue Development. Curr. Top. Dev. Biol..

[26] Hinton R.J. (2014). Genes that regulate morphogenesis and growth of the temporomandibular joint: A review. Dev. Dyn..

[27] Stocum D.L., Roberts W.E. (2018). Part I: Development and Physiology of the Temporomandibular Joint. Curr. Osteoporos. Rep..

[28] Liu X., Li N., Zhang H., Liu J., Zhou N., Ran C., Chen X., Lu Y., Wang X., Qin C. (2018). Inactivation of Fam20b in the neural crest-derived mesenchyme of mouse causes multiple craniofacial defects. Eur. J. Oral. Sci..

[29] Kozhemyakina E., Lassar A.B., Zelzer E. (2015). A pathway to bone: Signaling molecules and transcription factors involved in chondrocyte development and maturation. Development.

[30] Inada M., Wang Y., Byrne M.H., Rahman M.U., Miyaura C., López-Otín C., Krane S.M. (2004). Critical roles for collagenase-3 (Mmp13) in development of growth plate cartilage and in endochondral ossification. Proc. Natl. Acad. Sci. USA.

[31] Bechtold T.E., Kurio N., Nah H.-D., Saunders C., Billings P.C., Koyama E. (2019). The Roles of Indian Hedgehog Signaling in TMJ Formation. Int. J. Mol. Sci..

[32] Foulcer S.J., Nelson C.M., Quintero M.V., Kuberan B., Larkin J., Dours-Zimmermann M.T., Zimmermann D.R., Apte S.S. (2014). Determinants of Versican-V1 Proteoglycan Processing by the Metalloproteinase ADAMTS5. J. Biol. Chem..

[33] Melrose J., Shu C., Whitelock J.M., Lord M.S. (2016). The cartilage extracellular matrix as a transient developmental scaffold for growth plate maturation. Matrix Biol..

[34] Chen Y., Mehmood K., Chang Y.F., Tang Z., Li Y., Zhang H. (2023). The molecular mechanisms of glycosaminoglycan biosynthesis regulating chondrogenesis and endochondral ossification. Life Sci..

[35] Chen X., Li N., Hu P., Li L., Li D., Liu H., Zhu L., Xiao J., Liu C. (2023). Deficiency of Fam20b-Catalyzed Glycosaminoglycan Chain Synthesis in Neural Crest Leads to Cleft Palate. Int. J. Mol. Sci..

[36] Long F., Ornitz D.M. (2013). Development of the endochondral skeleton. Cold Spring Harb. Perspect. Biol..

[37] Fan M., Geng N., Li X., Yin D., Yang Y., Jiang R., Chen C., Feng N., Liang L., Li X. (2024). IRE1α regulates the PTHrP-IHH feedback loop to orchestrate chondrocyte hypertrophy and cartilage mineralization. Genes. Dis..

[38] Yang J., Andre P., Ye L., Yang Y.-Z. (2015). The Hedgehog signalling pathway in bone formation. Int. J. Oral. Sci..

[39] Mizuhashi K., Ono W., Matsushita Y., Sakagami N., Takahashi A., Saunders T.L., Nagasawa T., Kronenberg H.M., Ono N. (2018). Resting zone of the growth plate houses a unique class of skeletal stem cells. Nature.

[40] Mis E.K., Liem K.F., Kong Y., Schwartz N.B., Domowicz M., Weatherbee S.D. (2014). Forward genetics defines Xylt1 as a key, conserved regulator of early chondrocyte maturation and skeletal length. Dev. Biol..

[41] Matsushita Y., Chu A.K.Y., Tsutsumi-Arai C., Orikasa S., Nagata M., Wong S.Y., Welch J.D., Ono W., Ono N. (2022). The fate of early perichondrial cells in developing bones. Nat. Commun..

[42] Au T.Y.K., Yip R.K.H., Wynn S.L., Tan T.Y., Fu A., Geng Y.H., Szeto I.Y.Y., Niu B., Yip K.Y., Cheung M.C.H. (2023). Hypomorphic and dominant-negative impact of truncated SOX9 dysregulates Hedgehog-Wnt signaling, causing campomelia. Proc. Natl. Acad. Sci. USA.

[43] Deng Q., Li P., Che M., Liu J., Biswas S., Ma G., He L., Wei Z., Zhang Z., Yang Y. (2019). Activation of hedgehog signaling in mesenchymal stem cells induces cartilage and bone tumor formation via Wnt/β-Catenin. eLife.

[44] Mak K.K., Chen M.H., Day T.F., Chuang P.T., Yang Y. (2006). Wnt/beta-catenin signaling interacts differentially with Ihh signaling in controlling endochondral bone and synovial joint formation. Development.

[45] Wu J., Tian Y., Han L., Liu C., Sun T., Li L., Yu Y., Lamichhane B., D’Souza R.N., Millar S.E. (2020). FAM20B-catalyzed glycosaminoglycans control murine tooth number by restricting FGFR2b signaling. BMC Biol..

[46] Koosha E., Brenna C.T.A., Ashique A.M., Jain N., Ovens K., Koike T., Kitagawa H., Eames B.F. (2024). Proteoglycan inhibition of canonical BMP-dependent cartilage maturation delays endochondral ossification. Development.

[47] Mackie E.J., Tatarczuch L., Mirams M. (2011). The skeleton: A multi-functional complex organ. The growth plate chondrocyte and endochondral ossification. J. Endocrinol..

[48] Jing Y., Zhou X., Han X., Jing J., von der Mark K., Wang J., de Crombrugghe B., Hinton R.J., Feng J.Q. (2015). Chondrocytes Directly Transform into Bone Cells in Mandibular Condyle Growth. J. Dent. Res..

[49] Jing Y., Wang Z., Li H., Ma C., Feng J. (2020). Chondrogenesis Defines Future Skeletal Patterns Via Cell Transdifferentiation from Chondrocytes to Bone Cells. Curr. Osteoporos. Rep..

[50] Yang L., Tsang K.Y., Tang H.C., Chan D., Cheah K.S. (2014). Hypertrophic chondrocytes can become osteoblasts and osteocytes in endochondral bone formation. Proc. Natl. Acad. Sci. USA.

[51] Aghajanian P., Mohan S. (2018). The art of building bone: Emerging role of chondrocyte-to-osteoblast transdifferentiation in endochondral ossification. Bone Res..

[52] Long F., Qin X., Jiang Q., Nagano K., Moriishi T., Miyazaki T., Komori H., Ito K., Mark K.v.d., Sakane C. (2020). Runx2 is essential for the transdifferentiation of chondrocytes into osteoblasts. PLoS Genet..

[53] Rim Y.A., Nam Y., Ju J.H. (2020). The Role of Chondrocyte Hypertrophy and Senescence in Osteoarthritis Initiation and Progression. Int. J. Mol. Sci..

[54] Roughley P.J., Mort J.S. (2014). The role of aggrecan in normal and osteoarthritic cartilage. J. Exp. Orthop..

[55] Xiao Z.F., Su G.Y., Hou Y., Chen S.D., Lin D.K. (2018). Cartilage degradation in osteoarthritis: A process of osteochondral remodeling resembles the endochondral ossification in growth plate?. Med. Hypotheses.

[56] Hunter D.J., Bierma-Zeinstra S. (2019). Osteoarthritis. Lancet.

[57] Librizzi M., Naselli F., Abruscato G., Luparello C., Caradonna F. (2023). Parathyroid Hormone Related Protein (PTHrP)-Associated Molecular Signatures in Tissue Differentiation and Non-Tumoral Diseases. Biology.

[58] Tian Y., Ma P., Liu C., Yang X., Crawford D.M., Yan W., Bai D., Qin C., Wang X. (2015). Inactivation of Fam20B in the dental epithelium of mice leads to supernumerary incisors. Eur. J. Oral. Sci..

[59] Chai Y., Jiang X., Ito Y., Bringas P., Han J., Rowitch D.H., Soriano P., McMahon A.P., Sucov H.M. (2000). Fate of the mammalian cranial neural crest during tooth and mandibular morphogenesis. Development.

[60] Gu S., Wu W., Liu C., Yang L., Sun C., Ye W., Li X., Chen J., Long F., Chen Y. (2014). BMPRIA mediated signaling is essential for temporomandibular joint development in mice. PLoS ONE.

