# Supplementary material for: FAM20B-Catalyzed Glycosylation Regulates the Chondrogenic and Osteogenic Differentiation of the Embryonic Condyle by Controlling IHH Diffusion and Release
Source: Int J Mol Sci. 2025 Apr 24;26(9):4033. doi: 10.3390/ijms26094033 (PMC12071210; doi:10.3390/ijms26094033)
Supplement: Supplementary file 1 [file ijms-26-04033-s001.zip › ijms-3512923-supplementary.pdf]

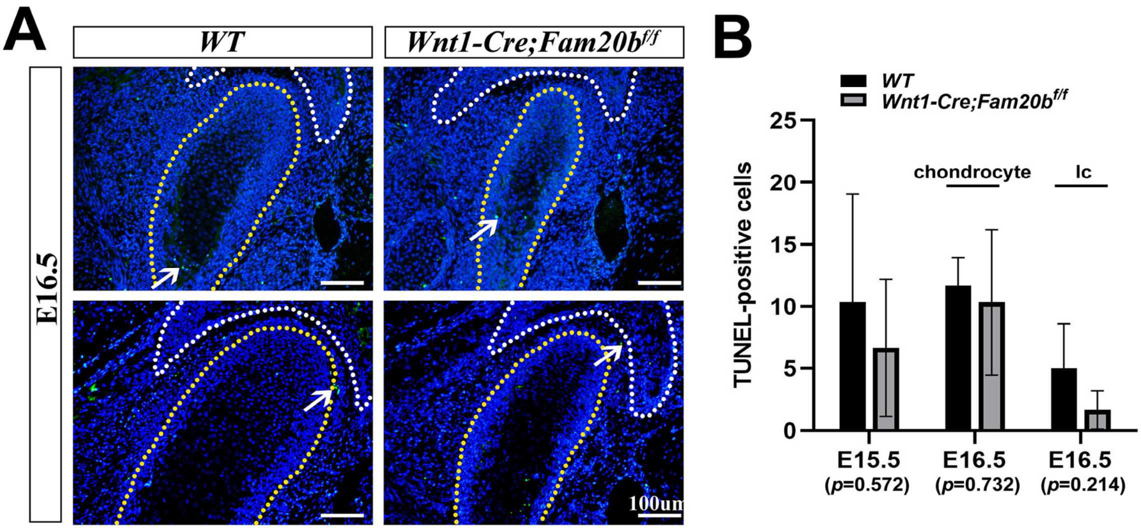

Supplementary Figure S1. Statistical analysis of apoptotic cells in TMJ. (**A**) TUNEL assay shows the apoptotic cells in E15.5 and E16.5 WT and *Wnt1-Cre;Fam20b^f/f^* TMJ. The white dotted lines outline the condyle and glenoid fossa. The white arrows indicate TUNEL-positive apoptotic cells. (**B**) Statistical assay of apoptotic cells in condyle chondrocytes and lower joint cavity, respectively. Data are represented as mean ± SD, E15.5 condyle chondrocytes (10 ± 8 cells vs. 6 ± 5 cells, p = 0.572), E16.5 condyle chondrocytes (11 ± 2 cells vs. 10 ± 6 cells, p = 0.732), and E16.5 lower joint cavity (5 ± 3 cells vs. 2 ± 2 cells, *p* = 0.214); n = 3; lc, lower joint cavity; scale bars, 100 μm.

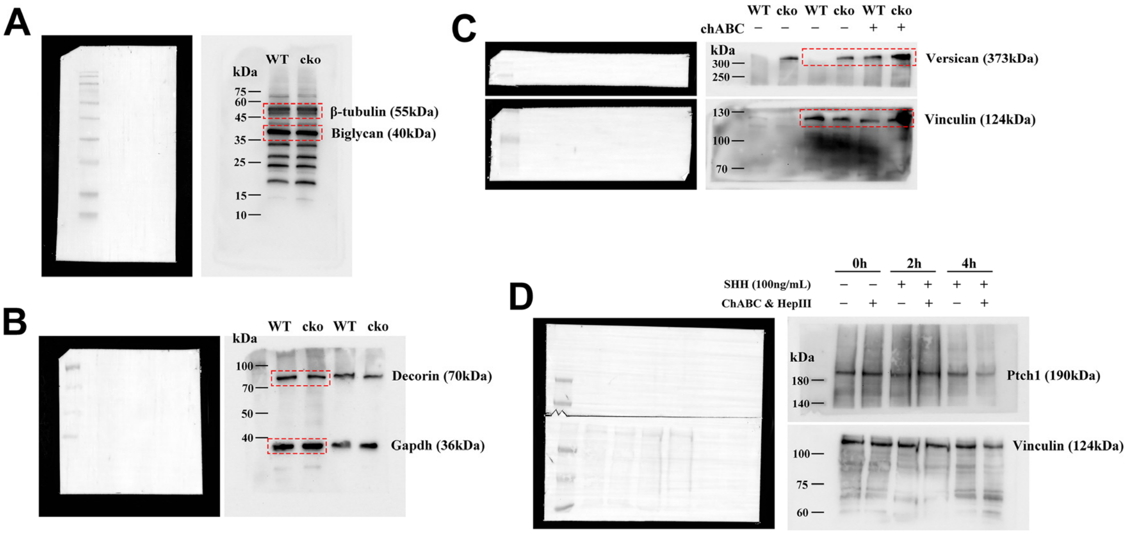

Supplementary Figure S2. The original data of the Western blots. (**A**) Non-cropped images of Western blot for Biglycan (mainly adhering CS chains) in WT and *Wnt1-Cre;Fam20b^f/f^* condyles. Β-tubulin was used as the standardized internal reference. (**B**) Non-cropped images of Western blot for Decorin (mainly adhering CS chains) in WT and *Wnt1-Cre;Fam20b^f/f^* condyles. Gapdh was used as the standardized internal reference. (**C**) Non-cropped images of Western blot for Versican (mainly adhering CS chains) in WT and *Wnt1-Cre;Fam20b^f/f^* condyles. Vinculin was used as the standardized internal reference. "+" represents that the protein was treated with chondroitinase ABC. The red dashed boxes in A-C represent the selected sections presented in the paper. (**D**) Non-cropped images of Western blot of Ptch1 protein levels in control and chondroitinase ABC- and heparinase III-treated mandibular condylar chondrocytes after 0 h, 2 h, and 4 h incubation with SHH growth factor addition. Vinculin was used as the standardized internal reference.
